# Vitamin D and subsequent all-age and premature mortality: a systematic review

**DOI:** 10.1186/1471-2458-13-679

**Published:** 2013-07-24

**Authors:** Lynne Rush, Gerry McCartney, David Walsh, Daniel MacKay

**Affiliations:** 1 NHS Greater Glasgow and Clyde, G12 0XH, Scotland; 2 NHS Health Scotland, Glasgow G2 2AF, Scotland; 3 Glasgow Centre for Population Health, Glasgow G2 4DL, Scotland; 4 Institute of Health and Wellbeing, University of Glasgow, Glasgow G12 8QQ, Scotland

**Keywords:** Vitamin D, 25OHD, Premature mortality, Systematic review, Meta-analysis

## Abstract

**Background:**

All-cause mortality in the population < 65 years is 30% higher in Glasgow than in equally deprived Liverpool and Manchester. We investigated a hypothesis that low vitamin D in this population may be associated with premature mortality via a systematic review and meta-analysis.

**Methods:**

Medline, EMBASE, Web of Science, the Cochrane Library and grey literature sources were searched until February 2012 for relevant studies. Summary statistics were combined in an age-stratified meta-analysis.

**Results:**

Nine studies were included in the meta-analysis, representing 24,297 participants, 5,324 of whom died during follow-up. The pooled hazard ratio for low compared to high vitamin D demonstrated a significant inverse association (HR 1.19, 95% CI 1.12-1.27) between vitamin D levels and all-cause mortality after adjustment for available confounders. In an age-stratified meta-analysis, the hazard ratio for older participants was 1.25 (95% CI 1.14-1.36) and for younger participants 1.12 (95% CI 1.01-1.24).

**Conclusions:**

Low vitamin D status is inversely associated with all-cause mortality but the risk is higher amongst older individuals and the relationship is prone to residual confounding. Further studies investigating the association between vitamin D deficiency and all-cause mortality in younger adults with adjustment for all important confounders (or using randomised trials of supplementation) are required to clarify this relationship.

## Background

The extent to which vitamin D deficiency may be important in explaining morbidity and mortality has recently been a focus for research. The importance of vitamin D for bone health and the prevention of rickets is well-established; however, observational data suggest that low levels are also associated with increased incidence of chronic diseases including cardiovascular disease, cancer, type II diabetes and multiple sclerosis [1-8]. Four meta-analyses of observational studies have also found an association with increased all-cause mortality [9-12]. Most of the populations in the included studies were elderly, limiting applicability to the wider population. It is also possible that the associations between vitamin D and mortality seen in observational studies are due to confounding with, for example, obesity or reduced physical activity causing both reduced vitamin D and negative health outcomes. In addition, observational associations may be due to reverse causality, whereby vitamin D is reduced as a consequence of disease processes. Those randomised controlled trials of vitamin D supplementation that are available are largely confined to elderly populations with pre-existing morbidity, for example, following hip fracture and are therefore not generalisable to either younger or pre-morbid populations.

The potential for vitamin D deficiency to explain higher mortality, and in particular premature mortality, is particularly relevant in Scotland. Vitamin D deficiency has been postulated as one of many explanations for the ‘excess’ mortality (that is, the higher levels of mortality not explained in terms of socio-economic circumstances) seen in Scotland, particularly in Glasgow, compared to the rest of the UK [13-18]. The vitamin D hypothesis is supported by evidence that deficiency is higher in Scotland than in the rest of the UK and the links in observational studies between low vitamin D levels and all age mortality and various chronic diseases [19].

Walsh et al. compared mortality data for Glasgow, Liverpool and Manchester, three cities with comparable histories of deindustrialisation, and with strikingly similar socio-economic profiles [20]. After adjustment for any remaining differences in levels of area deprivation, all-cause mortality was found to be 15% higher in Glasgow. Premature mortality (defined as deaths under 65 years) was 30% higher, and higher still for ages 15–45 years. Further evidence regarding the link between vitamin D deficiency and premature mortality is therefore required to evaluate whether the relatively high Scottish mortality rates may be in part explained by vitamin D deficiency.

This study aims to establish whether there is evidence of an association between vitamin D deficiency and premature mortality, and the potential for any association to be due to confounding.

## Methods

The systematic review was conducted according to the guidelines set out in the PRISMA statement [21].

### Inclusion criteria

Studies in all settings and countries and involving both male and female participants were included. Although the primary association of interest was the association between vitamin D and premature mortality, no upper age limit was specified in order that patterns of association at different ages could be identified. As the association of interest was between vitamin D and mortality in the general population, studies in which participants were selected on the basis of pre-existing illness were excluded. No restrictions were imposed for language, year of publication or duration of follow-up.

Randomised controlled studies, non-randomised controlled studies and cohort studies were included if they involved an appropriate population. Case–control, cross-sectional, ecological and case series studies were excluded, as they were considered to be at high risk of confounding. Studies which investigated the effects of vitamin D supplementation but which did not report vitamin D status, or which estimated vitamin D status on the basis of reported sun exposure or dietary intake were excluded, as these were felt likely to lack precision. Only studies that reported on all-cause mortality were included.

### Search strategy

Ovid Medline, Embase, Web of Science and the Cochrane library were searched until February 2012. The search terms used are detailed in Additional file 1. Duplicate results were removed using the reference management system Refworks. Screening of search results on title, or title and abstract, were carried out independently by two reviewers. Disagreements were resolved through discussion and reference to the inclusion criteria. Where articles were available only in abstract form, supplementary data were requested from the authors by email.

In order to identify relevant grey literature, the same search terms were entered in the British Library online catalogue and the internet search engines Google and Google scholar were searched using the terms “vitamin D” and “mortality”. The first 400 results from each were also screened independently by two reviewers.

Reference lists of relevant systematic reviews identified were screened for additional articles. The journals in which the main relevant studies were published were hand searched for previously unidentified articles (Additional file 1). Authors of the studies taken forward for critical appraisal and of the main relevant systematic reviews were contacted with requests for relevant unpublished data or research in progress.

For studies published in languages other than English, an internet translation tool was used to facilitate screening. If further information was required, the author was contacted with a request for translated material.

### Critical appraisal

Studies which met the inclusion criteria were assessed for bias and confounding using questions based on the Critical Appraisal Skills Programme (CASP) tool for appraisal of observational studies (Additional file 1) [22].

Critical appraisal was carried out independently by two of three reviewers, blinded to the other’s results. Where the recruitment methods or response rates within a study were unclear, the protocol for the original cohort study was accessed. Disagreements between the reviewers were resolved by discussion.

### Data extraction

The following data were extracted from the included studies by one reviewer and checked by another and recorded in a Microsoft Excel spreadsheet: year of publication; summary mortality measure and confidence interval; study size; method used to quantify vitamin D levels; average age of participants; average follow-up time; mean 25OHD level within each category or quantile; and the confounding variables adjusted for. Through a combination of prior awareness and insight gained while undertaking the literature review, we considered that the most likely confounders in the relationship between 25OHD levels and all-cause mortality were age, sex, ethnicity, season of measurement, smoking, physical activity, BMI, socioeconomic status, chronic disease, use of vitamin supplements and sun exposure. ‘Average’ follow-up time and age refers to the mean or median values, as reported in the individual studies.

### Analysis

As an initial descriptive analysis, we produced a scatterplot of log hazard ratio for all-cause mortality against the average 25OHD in each quantile or category for each of the included studies. We then performed a meta-analysis using Stata (v12.0) using a random effects model to account for heterogeneity between studies. In the included studies, hazard ratios for all-cause mortality for different quantiles or categories of vitamin D were compared to a reference group. The participants within each category were treated as distinct study populations and the hazard ratios entered separately into the meta-analysis.

A meta-analysis of the log transformations of the hazard ratios was carried out using the ‘metan’ command within Stata [23,24]. Separate forest plots summarising the effect sizes of the individual studies and the pooled effect sizes were obtained for the adjusted and unadjusted hazard ratios. In order to assess whether the association between 25OHD level and mortality was different in older versus younger participants, a stratified analysis was performed, producing separate pooled effect sizes for studies with an average participant age under and over 65 years. Previous research has suggested that follow-up time may influence the ability to detect significant effects between 25OHD and mortality, therefore a stratified analysis was performed to assess the influence of follow-up time on the effect size [25].

The standard error of the log hazard ratios was calculated and a funnel plot was produced using the ‘metafunnel’ command to assess for publication bias [26]. A more formal investigation of funnel plot asymmetry due to small study effects was also performed using Egger’s linear regression method, generated by the ‘metabias’ command to test whether there was a linear association between the effect size and its standard error [27].

## Results

### Search results

**Figure 1:**
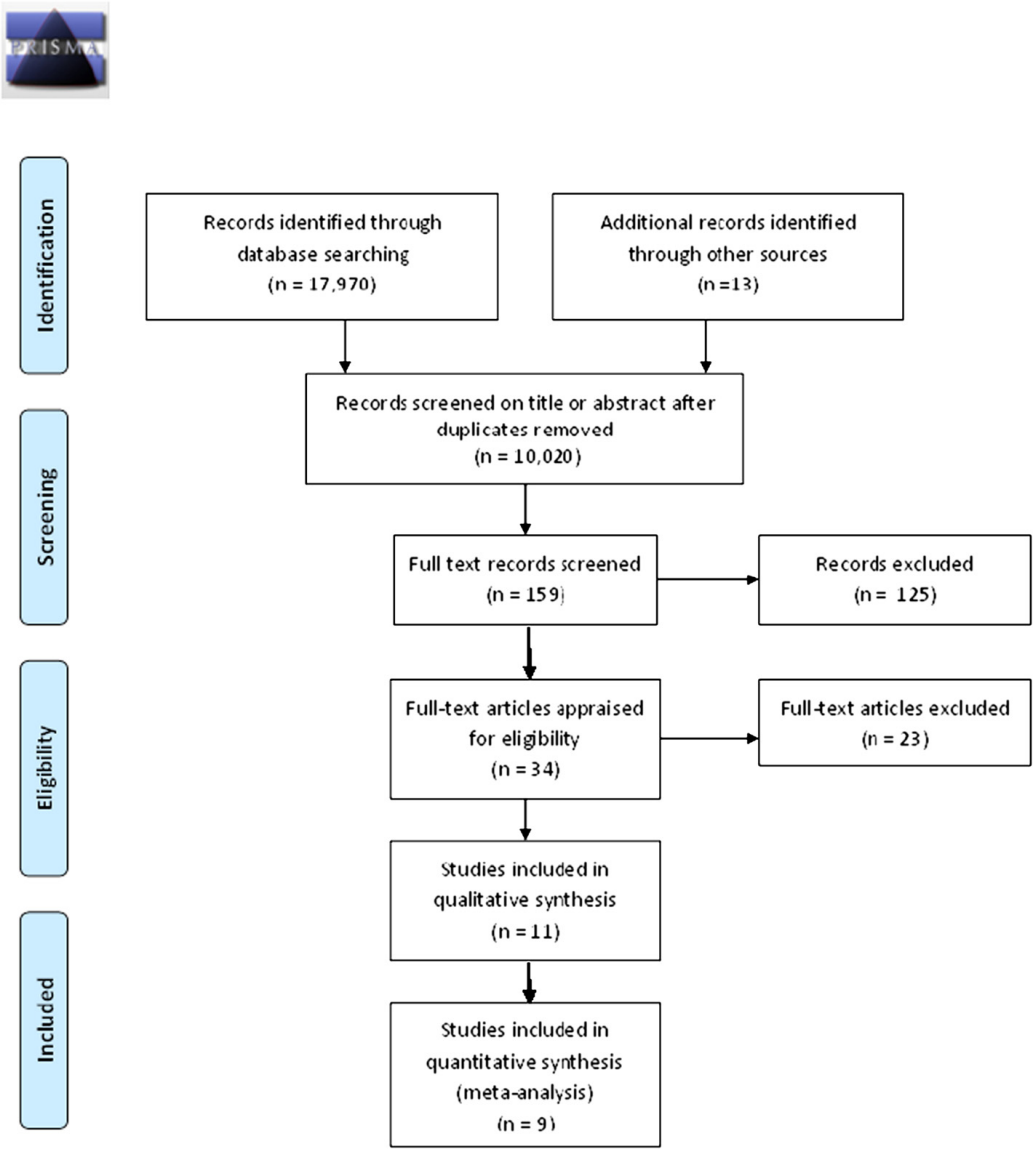
PRISMA 2009 Flow Diagram.

The PRISMA flow chart in Figure 1 summarises the results of the search process. The authors of the 11 articles that were available in abstract form only were contacted via email with a request for further information. One author could not be reached (mail undelivered). Of a further 11 authors contacted, nine responded. Four of these authors provided full-text versions of their papers, yielding a further three articles suitable for critical appraisal. Two articles were unobtainable either in abstract or full-text format. The articles that were unobtainable or available as abstracts only are listed in Additional file 1.

The breakdown of reasons for exclusion of full-text articles is detailed in Additional file 1. Of the 34 studies that were critically appraised, 23 were excluded. Additional file 1 lists the reasons for exclusion.

### Characteristics of studies included in the systematic review

Eleven studies were included in the review. Of these, nine were suitable for inclusion in the meta-analysis, containing 24,297 participants. 5,324 deaths occurred during follow-up. Table 1 lists the characteristics of the included studies.

**Table 1:** Characteristics of the studies included in the systematic reviews

**Study**	**Year**	**Country**	**Design**	**Population**	**Sample size**	**Average age (years)**^**a**^	**Response rate%**	**Overall representativeness of population**^**b**^	**Average follow-up duration (years)**^**a**^
Bates [28]	2011	UK	Prospective cohort	Men and women aged ≥65y	1,054	Males 75.8 Females 77.3	36	Fair	13.5
Ford [29]	2011	USA	Prospective cohort	Men and women aged over 20 across USA	7,531	Mean 45–46 (by ascending 25OHD concentration)	80 (2001/2 sample), 76 (2003/4 sample)	Excellent	Median 3.8
Ginde [30]	2009	USA	Prospective cohort	Men and women aged over 65 across USA	3,408	Mean 72–76 (by ascending 25OHD category)	60.1	Good	Median 7.3
Hutchinson [31]	2010	Norway	Prospective cohort	Men and women over age of 25 living in city of Tromso	Smokers – 2,410 Non-smokers – 4,751	Smokers – mean 57–58 (by ascending 25OHD quartile).	78	Good	Mean Smokers – 11.4 Non-smokers 11.8
						Non-smokers mean 59–61 (by ascending 25OHD quartile)			
Jia [32]	2007	UK	Prospective cohort	Men and women over age of 75 living in city of Aberdeen	398	Median 80	Men 25 Women 17	Fair	Median 5.95
Johansson [33]	2011	Sweden	Prospective cohort	Men aged 70–81 years living in cities of Gothenburg, Malmo and Uppsala	3,014	Mean 75	45	Fair	6
Melamed [34]	2008	USA	Prospective cohort	Men and women aged over 20 across USA	13,331	Mean 41.0-46.7 (by 25OHD quartile)	67.7	Excellent	Median 8.7
Michaelsson [35]	2010	Sweden	Prospective cohort	Men born during 1920–4 living in city of Uppsala	1,194	Mean 71	73	Good	Median 12.7
Semba [36]	2010	Italy	Prospective cohort	Men and women over 65 living in Tuscany	1,155	Median 71–78 (by ascending 25OHD quartile)	90.3	Good	6.5
Virtanen [37]	2011	Finland	Prospective cohort	Middle aged men and women in Eastern Finland	1,136	Mean 61–62 (by ascending 25OHD tertile)	Men – 85.6 Women – 78.4	Good	9.1
Visser [38]	2006	Netherlands	Prospective cohort	Men and women aged over 65 living in city of Amsterdam	1,260	No average given, all over 65, recalculated as mean 70.2-78.5 by category	81.7	Good	6

a – mean or median specified where reported in original study.

b - based on recruitment method and response rate.

The majority of the included studies reported mortality outcomes in the form of relative hazard ratios for different quantiles or categories of 25OHD. Johansson et al’s study did not fully report mortality outcomes or confidence intervals for different 25OHD quantiles while the study by Melamed et al. presented the outcome as a mortality rate ratio, of which was incompatible with the hazard ratios used in the other studies [33,34]. These studies are included in the narrative review but were excluded from the meta-analysis. In all but one of the other studies the quantile or category containing the highest 25OHD levels were used as the reference group; instead, Michaelsson et al. used the participants with 25OHD in the middle 10-90th centiles as a reference group [35]. The hazard ratios for the participants in the 0-5th and 0-10th centiles were entered into the meta-analysis but the hazard ratios for those in the upper categories (90th-100th centile and 95th-100th centile) were omitted. This ensured that all the hazard ratios entered into the meta-analysis measured effects in the same direction, i.e. comparison of mortality outcomes in individuals with lower compared to higher vitamin D levels within the same study population. Bates et al’s study reported the hazard ratio for all-cause mortality for a continuous increase in 25OHD only; Chris Bates and his colleague Gita Mishra kindly recalculated the hazard ratios for quartiles of 25OHD in order that this study could be included in the meta-analysis [28,39].

Two studies (Melamed and Ginde) presented data from the same cohort study. Ginde’s study was included in the meta-analysis, Melamed’s study having been already excluded [30].

### Relationship between 25OHD level and all-cause mortality

Significantly raised hazard ratios for all-cause mortality were seen at least in the lowest category or quantile of 25OHD compared to the reference category in all of the studies. However, in the fully adjusted models, three of the nine studies (Ford, Jia and Visser) did not find evidence of a significant association between 25OHD and all-cause mortality. No consistent differences were noted in the confounding factors adjusted for by these studies compared to the others.

**Figure 2:**
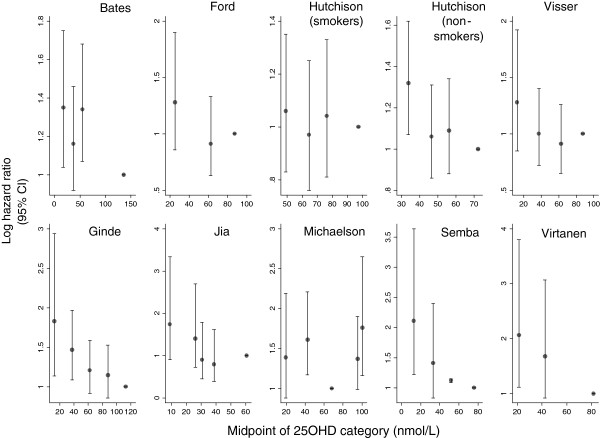
Scatterplot matrix of relationship between 25OHD category and all-cause mortality by study.

The relationships between 25OHD and all-cause mortality in the individual studies are displayed visually in the scatterplot matrix in Figure 2.

The fact that all but one of the studies used the highest category or quantile as the reference category suggests an a priori assumption that the true relationship between 25OHD and all-cause mortality is inverse; however, such a pattern is not seen consistently across the studies. In fact, a non-linear relationship is seen in six of the nine studies. In four of these studies, all-cause mortality actually appears to be reduced in categories where 25OHD is lower than in the reference group (Ford, Hutchinson (smokers), Jia and Visser). Michaelsson et al’s study is the only one in which all-cause mortality at higher levels of 25OHD is compared to more moderate levels. In this study, mortality is significantly elevated at both below the 10th and above the 95th centiles, with the 10-90th centiles as the reference group.

No consistent pattern was seen between the studies containing younger participants compared to those with an older population.

### Synthesis of results

**Figure 3:**
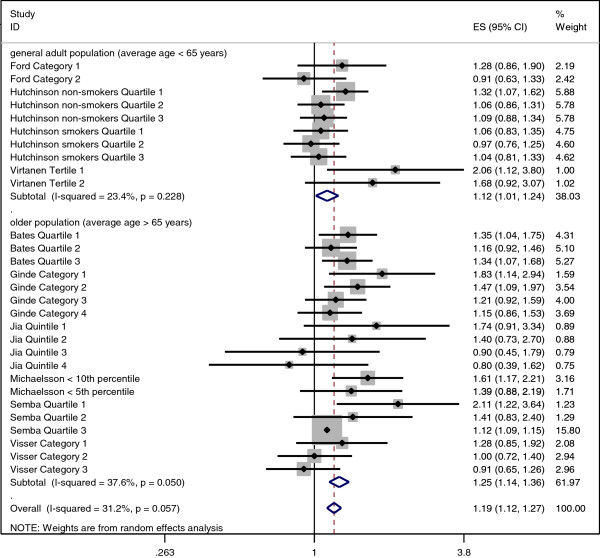
Forest plot displaying pooled effect of fully adjusted hazard ratios, stratified by age category.

Using the unadjusted hazard ratios, the overall effect size for all-cause mortality for the lowest quantile or category of 25OHD compared to the reference category within each study was 1.42 (95% confidence interval 1.30-1.55). In the age-stratified analysis, the pooled effect size for participants under 65 years was 1.30 (95% confidence interval 1.16-1.46) and for those aged over 65 years was 1.50 (95% confidence interval 1.32-1.71) (Additional file 1). The overall effect size for all-cause mortality using the adjusted hazard ratios was 1.19 (95% confidence interval 1.12-1.27). Stratified by age, the overall effect size was 1.12 (95% confidence interval 1.01-1.24) for studies with participants with mean age under 65 years and 1.25 (95% confidence interval 1.14-1.36) for those over 65 years (Figure 3). No consistent pattern was seen for effect size according to duration of follow-up using the unadjusted or adjusted hazard ratios (Additional file 1).

### Publication bias

The funnel plot of the logHRs against their standard errors can be seen in Additional file 1. Asymmetry towards the lower left region of the funnel plot suggests a lack of smaller studies with negative effects (i.e. those that do not show a reduction in all-cause mortality with increasing 25OHD levels). This is supported by the results of Egger’s test, which gives a p value of 0.04 suggesting that small study publication bias cannot be excluded. Note that only three studies contained non-elderly participants which makes it difficult to assess for publication bias in this subgroup.

In order to identify which factors within the studies were associated with a larger effect size, a meta-regression analysis was performed. Methodology and results are available as webmaterials; of note, increasing age was associated with a larger hazard ratio though did not remain significant after adjustment for multiple comparisons. In addition, the studies were highly variable in their adjustment for potential confounders. The impact of adjustment for the various confounders on the overall effect size was also investigated via metaregression; adjustment for socioeconomic status and use of vitamin supplements were associated with a significant increase in the overall hazard ratio but did not remain significant in the multivariate analysis.

## Discussion

### Main finding of this study

The meta-analysis of 29 hazard ratios from nine studies demonstrates a statistically significant increase in the risk of all-cause mortality in individuals with low versus high 25OHD (HR 1.42, 95% confidence interval 1.30-1.55). After maximal adjustment for confounding factors, the hazard ratio was attenuated but remained significant (HR 1.19, 95% confidence interval 1.12-1.27). In the age-stratified meta-analysis, the risk of all-cause mortality was significantly increased in both older (HR 1.25, 95% confidence interval 1.14-1.36) and, to a lesser extent, in younger participants (HR 1.12, 95% confidence interval 1.01-1.24).

### What is already known on this topic

The findings of this meta-analysis are consistent with previous systematic reviews that identified an inverse association between 25OHD levels and mortality [9-12]. As in the review by Zittermann et al., this relationship was not observed at all concentrations and there appeared to be no consistent pattern regarding at which concentrations mortality was highest and lowest. For example, two of the studies showed increased mortality at quartiles 1 and 3, but not at quartile 2, suggesting non-linearity in the relationship. Only the study by Michaelsson et al. used participants with 25OHD levels in the mid-range as a reference group, and in this study a U-shaped association between 25OHD level and mortality was observed [35].

### What this study adds

As far as we are aware, this is the only systematic review and meta-analysis that has specifically investigated whether the apparent association between low vitamin D status and all-cause mortality is age-dependent. We performed a comprehensive search strategy including an extensive grey literature search, ensuring that all relevant information should have been identified. We adhered to strict inclusion criteria which ensured that only studies that were representative of the general population were included, which enhances the generalisability of our findings.

### Limitations of this study

Several limitations of the review are acknowledged**.** Few studies provided data for individuals aged less than 65 years and the results are based on aggregate data. Availability of individual level data would have allowed stratification of mortality outcomes by narrower age bands. The possibility of publication bias is also acknowledged (Additional file 1), although the funnel plot suggests that the unpublished studies are likely to be those in younger adults which do not support an inverse relationship between 25OHD level and mortality and inclusion of these studies would therefore not alter the conclusions of the meta-analysis.

All of the included studies were observational in design and thus have limited capacity to demonstrate causal associations between vitamin D levels and all-cause mortality, and do not inform on the question of whether a change in vitamin D (e.g. through supplementation) changes mortality outcomes. None of the included studies adjusted for all important confounding factors; for example, four of the 11 included studies made no adjustment for socioeconomic status making residual confounding in the association between low vitamin D and all-cause mortality likely.

## Conclusions

In this systematic review and meta-analysis, we sought to evaluate evidence of the association between vitamin D and premature mortality. Although a significant increase in all-cause mortality was found in study participants of all ages with low compared to higher 25OHD levels, the pooled effect size was lower for studies with participants with an average age of less than 65 years compared to the studies containing older participants. In the multivariate meta-regression analysis, increasing age was associated with a statistically significant increase in the predicted hazard ratio. Many of the included studies failed to adjust for important confounding factors such as socioeconomic status which makes residual confounding in the relationship likely. At present, there is insufficient data to conclude that there is an inverse association between low vitamin D status and overall mortality in younger adults. Further observational studies using younger participants and with adjustment for all important confounders (or alternatively, using randomised trials of supplementation) are required to clarify this relationship.

The nature of Scotland’s higher levels of premature mortality (compared to elsewhere in the UK) must also be considered when attempting to draw conclusions about the potential contribution of vitamin D deficiency. Although cardiovascular disease and cancer remain major contributors to Glasgow’s higher mortality relative to Liverpool and Manchester, the majority of the ‘excess’ deaths under the age of 65 years are related to alcohol and drugs, suicide and external causes (which include violence and accidents) [20]. Increasing recognition of the effects of vitamin D on cellular processes have generated many hypotheses regarding how deficiency may be implicated in the pathogenesis of cardiovascular disease, cancer and other conditions. It seems less likely however that a causal association between 25OHD levels and road traffic accidents or drug-related deaths could be considered plausible.

## Competing interests

The authors declare that they have no competing interests.

## Authors’ contributions

LR, GM and DW conceived of the study design. LR carried out the database search. LR, GM and DW screened the search results and critically appraised the relevant articles. DM provided advice and assistance with the meta-analysis. All of the authors approved the final manuscript.

## Pre-publication history

The pre-publication history for this paper can be accessed here:

http://www.biomedcentral.com/1471-2458/13/679/prepub

## Supplementary Material

Additional file 1 **Webmaterials **[40-42]. Click here for file
