# Supplementary material for: Vitamin D and subsequent all-age and premature mortality: a systematic review
Source: BMC Public Health. 2013 Jul 24;13:679. doi: 10.1186/1471-2458-13-679 (PMC3727990; doi:10.1186/1471-2458-13-679)
Supplement: Additional file 1 — Webmaterials [40-42]. [file 1471-2458-13-679-S1.docx]

**Webmaterials**

**Webappendix 1 Search strategy**

**Ovid MEDLINE (R) In-Process & Other Non-Indexed Citations and Ovid Medline (R) 1946-Present and Embase 1974-Present**

Search terms (searching as keyword, limit to human):

1 vitamin D OR vitamin D def* OR hypovitaminosis D OR calcifediol OR calcidiol OR 25-hydroxycholecalciferol OR 25-hydroxyvitamin D OR 25OHD

2 mort* OR death OR surviv* OR life expectancy

3 Combine 1 AND 2

**Web of Science**

Search terms (searching within topic)

vitamin D OR vitamin D def* OR hypovitaminosis D OR calcifediol OR calcidiol OR 25-hydroxycholecalciferol OR 25-hydroxyvitamin D OR 25OHD

AND

mort* OR death OR surviv* OR life expectancy

**The Cochrane Library**

(searching within title, abstract or keywords)

vitamin D OR vitamin D def* OR hypovitaminosis D OR calcifediol OR calcidiol OR 25-hydroxycholecalciferol OR 25-hydroxyvitamin D OR 25OHD

AND

mort* OR death OR surviv* OR life expectancy

**Webappendix 2 Journals hand searched for relevant articles** (searched from inception – present day)

International Journal of Epidemiology

American Journal of Epidemiology

Journal of Clinical Endocrinology and Metabolism

American Journal of Clinical Nutrition

European Journal of Nutrition

European Journal of Clinical Nutrition

Clinical Endocrinology

European Journal of Clinical Endocrinology

British Journal of Nutrition

**Webappendix 3 Articles unobtainable or available as abstract only**

**Unobtainable articles**

Vitamin D may help keep your heart strong. However, more studies are needed before a clear link can be established. *Heart advis.* 2008;11(11):4.

Gatto S, Gimigliano F, Gimigliano R, Iolascon G. Prevention of falls and role of calcium and vitamin D. *Aging ClinExp Res.* 2011;23(2 Suppl):20-21.

**Articles available in abstract form only**

Schierbeck LL, Rejnmark L, Tofteng CL, Stilgren L, Eiken P, Mosekilde L, et al. Vitamin D and cardiovascular outcome in healthy postmenopausal women.American Heart Association's Scientific Sessions, Orlando, FL. 12 -16^th^ November 2011. *Circulation;* 2011;124 (21 suppl. 1)

Kritchevsky SB, Houston DK, Neiberg R, Tooze J, Hausman DB, Cauley JA, et al. Serum 25-Hydroxyvitamin D, Parathyroid Hormone and Mortality in Community-Dwelling Older Adults. *Gerontologist* 2010;50:218-218.

Liu L. Micronutrients, Inflammatory Biomarkers, and Risk of Cardiovascular Disease and All-Cause Mortality in the United States: Implications for a Healthier Diet and Longevity. *Circulation* 2010;122(2):E85-E86.

vonMuhlen D, Garland C, Bettencourt R, Barrett-Connor E. Are Serum Levels of 25-hydroxyvitamin D or 1,25-dihydroxyvitamin D Associated with Longevity? *Circulation*. 2009;119(10):E318-E318.

Cummings SR, Cawthon PM, Parimi N, Barrett-Connor E, Ensrud KE, Hoffman AR, et al. Low Vitamin D Levels and Risk of Death in Older Men: A Prospective Study. *J Bone Miner Res*. 2008;23:S114-S114.

Waren E, Christiansen C, Haug E, Rothenberg E, Mellstroem D. Decreasing Calcidiol (25-OHD3) In Serum Is Related to Impaired Function and Increased Mortality in The Elderly. *J Bone Miner Res.* 2008;23:S291-S291.

Varosy PD, Cummings SR, Hillier TA, Stone KL, Ensrud KE, Reid IR, et al. Vitamin D supplementation and risk of CHD death. *J Bone Miner Res*. 2006;21:S173-S173.

Formiga F, Ferrer A, Fraga A, Pujol R. Vitamin D levels and mortality of any cause in nonagenarians. NonaSantfeliu Study. *Med Clin* 2011;137(3):137-138.

Frost SA, Nguyen T, Bliuc D, et al. Effects of Vitamin D Deficiency and High Parathyroid Hormone on Mortality Risk in Elderly Men.Annual Meeting of the American Society for Bone and Mineral Research, 2010.Oral Presentations, Presentation Number 1168. Concurrent Oral Session 28: Vitamin D - Translational Studies. Available at: <http://medicineworks.com/vitamin-d-levels-linked-mortality-older-men-independent-age-and-level-parathyroid-hormone> (accessed 2^nd^ August 2012).

**Webtable 1 Critical appraisal checklist**

| What were the aims of the study and were they clearly stated?  Was the study design appropriate?  What method was used to recruit participants?  What exclusion criteria were applied?  What were the response and follow-up rates?  Was the sample representative of the general adult population?  What method was used to ascertain mortality outcomes?  What was the duration of follow-up?  Were the results presented appropriately?  Were the findings consistent with other studies?  Can the results be generalised to other populations?  What confounding factors were considered? |
| --- |

**Webtable 2 Reasons for exclusion of full-text articles prior to critical appraisal**

| Study population unsuitable (selected on basis of pre-existing illness or institutionalised) | 67 |
| --- | --- |
| No suitable 25OHD measurement or mortality outcome | 18 |
| Editorial/correspondence/review article | 22 |
| Abstract only available | 10 |
| Same mortality data contained within other study | 3 |
| Article unobtainable | 2 |
| Erroneous result i.e. entire journal cited without reference to specific article | 2 |
| Duplicate not otherwise identified | 1 |

**Webtable 3 Articles excluded after critical appraisal**

| Concerns over recruitment methods | 8 |
| --- | --- |
| Study population selected on basis of pre-existing morbidity | 6 |
| No mortality outcome | 1 |
| No 25OHD measurement reported | 1 |
| No all-cause mortality outcome | 2 |
| Results presented inappropriately for inclusion | 5 |

**Webfigure 1 Forest plot displaying pooled effect of unadjusted hazard ratios, stratified by age category**

**Webfigure 2 Forest plot displaying pooled effect of fully adjusted hazard ratios, stratified by average duration of follow-up**

**Webfigure 3 Funnel plot to assess possible publication bias, stratified by age category**

**Meta-regression analysis**

**Methods**

In order to explore the underlying reasons for heterogeneity between the included studies, a meta-regression analysis was performed using the ‘metareg’ command on Stata.^27^Univariate and multivariate analyses were carried out using the following variables: average age of participants; study duration; sample size; average value in 25OHD reference category; number of key confounding factors that were adjusted for in each study; and the method used to quantify 25OHD. Five of the covariates entered in the meta-regression analysis were continuous variables. Most of the studies reported the mean age within each quantile or category. Where this was not reported, the mean age of the overall study population was used. For the categorical variable (method), dummy variables were used; studies that used high performance liquid chromatography (HPLC) or mass spectrometry (MS) were assigned a value of 1 and studies using other methods were assigned a value of 0.

For the 25OHD reference category, the average value for 25OHD within the reference group was extracted as a summary measure. 25OHD values presented in ng/ml were converted to nmol/l by multiplying by 2.496.^36^ Where the reference category was presented as a range, the midpoint was calculated and used to represent the average value in this category. If the upper category was open-ended, the average value was approximated by taking the lowest value in the category plus two times the distance between this value and the midpoint of the previous category. This method of estimating an appropriate mid-point within open-ended categories for continuous data has been cited by other researchers.^40^

An adjustment for multiple testing was performed using the ‘permute’ command.^27^ This adjustment is based on Monte Carlo simulation and randomly reallocates the covariates to the outcomes many times, calculating multiple test statistics. The p value is amended according to the number of times the test statistic is equal to or greater than the observed statistic. The adjusted p value gives the probability of a test statistic for any of the covariates as extreme or more extreme than that of observed value under the null hypothesis that all of the regression coefficients are zero. The number of permutations was set at 20,000. This adjustment tends to produce slightly higher p values for each coefficient, reducing the likelihood that false positive results will be found by chance (type I error) as a result of multiple testing (but is less conservative than those produced by the alternative Bonferroni method).

The relationship between the average age of participants and the effect size was further explored by producing a bubble plot using the ‘graph, randomsize’ option, which shows the fitted regression line for average age of participants against the log hazard ratio. The size of the circles corresponds to the weight of each study in the meta-regression model, as determined by the inverse of its total variance.^27^ To further investigate the influence of the inclusion or exclusion of confounding variables, a separate univariate and multivariate analysis was then performed to identify whether adjustment for any of the following variables had a significant influence on the effect size: socioeconomic status, vitamin supplement use, ethnicity, sun exposure and chronic disease.

**Results of meta-regression analysis**

The overall heterogeneity in the fully adjusted studies was 31.2% (p = 0.06). Subgroup analyses demonstrated that heterogeneity was greater in the studies based on older participants (31.2%, p = 0.05) compared to those with a general adult population (23.4%, p = 0.23). The sources of heterogeneity were explored by performing a meta-regression analysis using the fully adjusted hazard ratios. Webtables 4 and 5 show the results of the univariate and multivariate analyses. The regression coefficient, 95% confidence intervals, p values and p values after adjustment for multiple testing are displayed. The method used to quantify 25OHD was significant in the univariate analysis, remaining so after adjustment for multiple testing. The average age of participants (mean or median, as previously) was significant in the multivariate model, but did not remain so after adjustment. The bubble plot in webfigure 6 displays the fitted regression line from the univariate analysis for average age of study participants and demonstrates the direction of the relationship, i.e. the expected increase in the logHR as age increases.

**Meta-regression analysis of individual study characteristics**

**Webtable 4 Univariate analysis**

| **Variable** | **Coefficient** | **95% confidence interval** | **Unadjusted p value** | **Multiplicity adjusted p value** |
| --- | --- | --- | --- | --- |
| Average age (years) | 1.00 | 0.99 – 1.10 | 0.05 | 0.06 |
| Average follow-up (years) | 1.00 | 0.99 – 1.02 | 0.31 | 0.37 |
| Sample size | 0.99 | 0.99 – 1.00 | 0.10 | 0.11 |
| Average 25OHD value of reference category | 1.00 | 0.99 – 1.00 | 0.85 | 0.90 |
| Number of confounding variables adjusted for | 1.05 | 0.99-1.10 | 0.06 | 0.06 |
| Method of vitamin D ascertainment (HPLC+/- MS vs other method) | 1.37 | 1.15-1.63 | 0.00 | 0.00 |

**Webtable 5 Multivariate analysis**

| **Variable** | **Coefficient** | **95% confidence interval** | **Unadjusted p value** | **Multiplicity adjusted p value** |
| --- | --- | --- | --- | --- |
| Average age (years) | 1.01 | 1.00-1.02 | 0.04 | 0.17 |
| Average follow-up (years) | 1.02 | 0.99-1.05 | 0.13 | 0.43 |
| Sample size | 1.00 | 0.99-1.00 | 0.27 | 0.71 |
| Average 25OHD value of reference category | 1.00 | 0.99-1.00 | 0.92 | 1.00 |
| No. of confounding variables adjusted for | 1.05 | 0.97-1.14 | 0.16 | 0.52 |
| Method (HPLC+/- MS vs other method) | 1.19 | 0.89-1.61 | 0.22 | 0.63 |

I²residual = 0.00% Adjusted R² = 100% Joint test for all covariates p = 0.0006

**Webfigure 4 Bubble plot of fitted regression line for average age of study participants**

Five of the key confounding factors were not adjusted for by all of the studies. These were: socioeconomic status, use of vitamin supplements, presence of chronic disease, sun exposure and ethnicity. In order to investigate whether inclusion or exclusion of these variables was a significant source of heterogeneity, a second meta-regression analysis was performed. Dummy variables were assigned to each study according to whether these variables had been adjusted for (1 = yes, 0 = no). The results of the univariate and multivariate analyses are presented in webtables 7 and 8. Adjustment for socioeconomic status and vitamin supplement use were significant in the univariate analysis and remained so after adjustment for multiple testing.

In the multivariate model, adjustment for socioeconomic status was significant, but not after the multiple adjustment. Adjustment for sun exposure, chronic disease or ethnicity was not significant in either model. It is noted that for sun exposure, the multiplicity adjusted p value is greater than the unadjusted value – studies that have investigated this method show that larger p values are usually, but not always, obtained by permutation than by meta-regression.^41^  This demonstrates that the use of a multiplicity adjustment does not mean that the possibility of finding spurious significant results through a combination of small number of studies and large number of covariates can be discounted.

**Meta-regression analysis of variation in confounding factors adjusted for in individual studies**

**Webtable 6 Univariate analysis**

| **Confounding variable adjusted for (1 yes, 0 no )** | **Coefficient** | **95% confidence interval** | **P value** | **Adjusted P value** |
| --- | --- | --- | --- | --- |
| Socioeconomic status | 1.17 | 1.00 – 1.34 | 0.02 | 0.03 |
| Vitamin supplement use | 1.18 | 1.02 – 1.37 | 0.01 | 0.04 |
| Sun exposure | 0.97 | 0.68 – 1.38 | 0.88 | 0.87 |
| Chronic disease | 0.93 | 0.78- 1.11 | 0.43 | 0.51 |
| Ethnicity | 1.04 | 0.88-1.23 | 0.58 | 0.63 |

**Webtable 7 Multivariate analysis**

| **Confounding variable** | **Coefficient** | **95% confidence interval** | **P value** | **Adjusted P value** |
| --- | --- | --- | --- | --- |
| Socioeconomic status | 1.21 | 1.00-1.47 | 0.04 | 0.22 |
| Vitamin supplement use | 1.10 | 0.89-1.35 | 0.36 | 0.70 |
| Sun exposure | 0.98 | 0.64-1.51 | 0.96 | 1.00 |
| Chronic disease | 0.84 | 0.68-1.03 | 0.11 | 0.33 |
| Ethnicity | 0.93 | 0.75-1.14 | 0.50 | 0.97 |

I²residual = 23.51% Adjusted R² = -56.3% Joint test for all covariates p = 0.0953

**Sensitivity analysis**

The results of the meta-regression suggest that the method used to quantify 25OHD has a significant influence on the effect size detected. A sensitivity analysis was performed, excluding the effects from the studies by Michaelsson et al and Virtanen et al, the two studies that used HPLC or MS, which are regarded as the most robust methods for 25OHD measurement.^42^  The revised hazard ratio was lower at 1.16 (95% confidence interval 1.09-1.23). In the age-stratified meta-analysis (excluding the same studies), the hazard ratios were 1.22 (95% confidence interval 1.12-1.33) in the studies with average age of participants over 65 years and 1.09 (95% confidence interval 1.00-1.19) in the studies with average age under 65 years.
